# Just how miserable is work? A meta-analysis comparing work and non-work affect

**DOI:** 10.1371/journal.pone.0212594

**Published:** 2019-03-05

**Authors:** Martin J. Biskup, Seth Kaplan, Jill C. Bradley-Geist, Ashley A. Membere

**Affiliations:** 1 Department of Psychology, George Mason University, Fairfax, VA, United States of America; 2 College of Business, University of Colorado, Colorado Springs, Colorado Springs, CO, United States of America; University of Warsaw, POLAND

## Abstract

Although we spend much of our waking hours working, the emotional experience of work, versus non-work, remains unclear. While the large literature on work stress suggests that work generally is aversive, some seminal theory and findings portray working as salubrious and perhaps as an escape from home life. Here, we examine the subjective experience of work (versus non-work) by conducting a quantitative review of 59 primary studies that assessed affect on working days. Meta-analyses of within-day studies indicated that there was no difference in positive affect (PA) between work versus non-work domains. Negative affect (NA) was higher for work than non-work, although the magnitude of difference was small (i.e., .22 *SD*, an effect size comparable to that of the difference in NA between different leisure activities like watching TV versus playing board games). Moderator analyses revealed that PA was relatively higher at work and NA relatively lower when affect was measured using “real-time” measurement (e.g., Experience Sampling Methodology) versus measured using the Day Reconstruction Method (i.e., real-time reports reveal a more favorable view of work as compared to recall/DRM reports). Additional findings from moderator analyses included significant differences in main effect sizes as a function of the specific affect, and, for PA, as a function of the age of the sample and the time of day when the non-work measurements were taken. Results for the other possible moderators including job complexity and affect intensity were not statistically significant.

## Introduction

*“This is the real secret of life -- to be completely engaged with what you are doing in the here and now*. *And instead of calling it work*, *realize it is play*.*”* - Philosopher Alan W. Watts

*“Oh*, *you hate your job*? *Why didn’t you say so*? *There’s a support group for that*. *It’s called everybody*, *and they meet at the bar*.*”* - Comedian Drew Carey

Paid work occupies a great deal of daily life for many people. On average, employed Americans work on about 60%-70% of their days and, on those days, spend about half their waking time working [1]. Given the amount of time we spend working, our well-being during that time is a matter of no small consequence, sometimes impacting decisions about whether, and how much, to work. The question of how the affective experience of work compares to that of non-work may be of increasing relevance today. This is due to the large numbers of baby-boomers reaching retirement age and making decisions as to whether/when to retire [2] and in light of the current societal shift towards the “gig economy” of freelance and independent contracting work arrangements [3]. The comparative experience of individuals’ well-being at work versus non-work also has potential implications for policy-makers who are interested in shaping strategies to promote societal well-being [4].

So, just how do people feel in work versus in non-work activities and locations? Anecdotal evidence (see contrasting quotes above), theoretical perspectives, and empirical studies suggest very different conclusions about the emotional experience of work. At one extreme, classical economic theories regard work as “disutility” - being an inherently unpleasant activity that individuals engage in solely for instrumental financial purposes [5]. Indeed, many definitions of “work” include the notion of *obligation* or “obligated time,” while “leisure” is characterized as the “antithesis of work, or “non-obligated time” [6]. This characterization of work as the less desirable contrast to non-work is also a prominent feature of scientific management, piece-rate compensation schemes [7], and popular accounts of work such as in Studs Terkel’s classic book “Working” [8].

In contrast to the above, some psychologists and sociologists have offered a very different perspective – making claim to the possible well-being *benefits* of work(ing). For instance, Jahoda posited that work provides people with various non-financial benefits (e.g., time structure, social contact) [9]. Hochschild presented data supporting the claim that in modern society, the workplace provides people with a sense of achievement and belonging, whereas home life has become increasingly stressful [10]. Similarly, Csikszentmihalyi and LeFevre noted “the paradoxical situation of people having many more positive feelings at work than in leisure” (p. 821) [11]. Furthermore, eudaimonic perspectives such as Self-Determination Theory highlight the benefits that work can provide in the form of personal growth, meaning, and intrinsic motivation [12].

### The primary study question and approach

In the current research, we examine the veracity of these different perspectives regarding the relative emotional experience of work versus non-work. To do so, we compare employees’ reported positive affect (PA) and negative affect (NA) regarding work versus non-work, both in terms of activity and location. Specifically, we conducted meta-analyses of within-person studies wherein people provide ratings of PA and/or NA *both* at, and away from, work. Using this strategy, mean levels in PA or NA would indicate differences in felt affect across domains. Additionally, we examine a number of moderators which allow for further insight into the puzzle behind the seemingly discrepant views outlined above on how people feel about/during/doing work. Indeed, a key impetus behind the current meta-analyses was that of testing for potential moderators which might explain *when*, *why*, and *for whom* affective experiences of work tend to be more (or less) favorable than non-work.

As such, we see this meta-analysis as making two primary contributions. First, it provides evidence about the relative role of work and non-work experiences with respect to psychological well-being. While a substantial body of work exists examining the interface of work and non-work domains and on affective spillover among those domains [13], few studies explicitly seek to directly compare the relative emotional experience of work versus non-work.

This oversight seems problematic as results regarding this issue can inform decision-making at various levels. At the individual level, results from the current study may guide decisions about how to spend one’s time (to the degree people have choice in doing so). As mentioned above, for an increasing number of people, work can be, in many ways, “optional” or discretionary, such as for some potential retirees [2]. Also, with a shift toward freelancing and other contingent (non-traditional) work arrangements comes increasing discretion with regard to what, when, where, and how people work [3].

At the governmental level, the comparative experience of well-being at work versus non-work has implications for policy aimed at promoting societal well-being [4]. To this point, discussions are underway by business and political leaders speculating as to the possible impacts of technological change and automation of work on the well-being of individuals and on the well-being of society. One possible policy-level approach to the much-feared mass displacement of workers by machines/automation [14], is that of a “basic income” which would be received by members of a society “no strings attached” - no work required. The idea - which has received favor from leaders both past and present, including the likes of Elon Musk, Richard Branson, and Milton Friedman - is, in fact, currently being tested in Finland in a real-world “social experiment” of sorts [14]. The Finnish government is providing a select number of residents with a basic income (amounting to approximately the equivalent of $16,000 annually) for two years and assessing the impact on recipients’ well-being and their decisions about how to spend their time if no longer “required” to earn money to live [14].

Beyond addressing the question of whether work is "better" or "worse" than non-work, a second intended contribution of the current study derives from its ability to explore moderators which speak to potentially important conceptual and methodological issues in the work and the affect literatures. For example, from a theoretical perspective, these findings can provide insight about the relative importance of different purported contributors to psychological benefits of work. For instance, one moderator we address is whether there are differences in work versus non-work affect when work is measured/recorded as a location (i.e., the workplace) versus as an activity (i.e., working). Findings that people feel relatively better (i.e., higher PA/lower NA) when work is an *activity* (as compared to work as *location*) would suggest that psychological benefits of work largely owe to task-related processes and phenomena such as cognitive busyness [9] and goal progress [15]. Conversely, the opposite finding would imply that non-task elements of work (e.g., socializing with colleagues or simply being away from home) are responsible for these benefits. We describe other examples in the following pages.

To investigate these questions, we incorporated studies that used methodologies seeking to capture/approximate experiential affect (as opposed to one-time recall/summary affect reports). Specifically, we included studies using one of two different methodologies - experience sampling methodology (ESM) or the day reconstruction method (DRM) [16]. In studies using real-time methods (ESM), employees report their current affect (e.g., *how do you feel right now*?) throughout the day [17]. In DRM studies, individuals reconstruct the previous day by listing the time and content of their activities and then reporting how they felt during each episode [16] These methodologies are now prevalent in psychology and management research, partly supplanting one-time affect assessments [17].

An alternative approach to addressing the main questions would have been to compare one-time, overall assessments of how individuals feel “in general” about work and non-work. However, these assessments capture psychological constructions or overall evaluations of well-being (e.g., about domains) rather than the moment-to-moment affective experiences of interest here [18, 19]. Owing to these differences in terms of construct representation and response process [18, 20], the relative reported favorability of a particular activity can vary dramatically using episodic versus one-time evaluative measurements. As an illustration of this phenomenon, individuals report “interacting with one’s children” as being very enjoyable *in general*, yet report *specific episodes* of interaction with one’s children as among the lowest/worst activities in terms of positive affect [16]. As others have noted, no given method to assess well-being is unequivocally “superior” to other methods, *per se*; rather, the phenomenon/question of interest should guide the choice among them [18]. Given the current focus on the real-time emotional phenomenology of these domains, including studies incorporating episodic measurements of affect seemed most appropriate.

Returning to the main research question as to whether and how affective experiences of work compare with non-work affective experience, the existing evidence seems to paint a somewhat mixed picture. Consistent with the notion that work provides various psychological benefits [9, 21] and represents a reprieve from home life [10], some studies seem to cast work in a favorable light. For example, Snir and Zohar found positive affect at work to be at least as high as during leisure activities [22]. Another study showed that work was more “exciting” than leisure [23]. Still other findings (many of which were not focused on the affective experience of work) indicate that work affect is more *similar to* - versus *distinct/different from* - affect in other life domains [24]. These findings call into question the notion that work is a less favorable affective experience than non-work.

On the other hand, several large-scale studies suggest a more decidedly negative role for work in terms affect. For example, several DRM studies have found that affective well-being at work is among the lowest of the various activities for which people reported their affect [16, 25–27]. Also, in a recent, very large ESM investigation of approximately 1.5 million observations from 20,000 individuals, the researchers reported that work ranked *second lowest* in happiness among 39 daily activities; the activity with the lowest happiness rating being “sick in bed” [28]. Results such as these perhaps suggest that the studies noted above showing work as relatively more favorable are anomalies, possibly owing to smaller sample sizes or study-specific features. Indeed, what seemingly makes those findings and also ideas like Hochschild’s Time Bind [10] and the concept of “flow” at work [11] intriguing is that they contradict the intuitive, common-wisdom notion of work as disutility. Taken together then, the available evidence is somewhat mixed (prompting this meta-analysis) but appears to favor the prediction that people tend to report higher PA and lower NA for non-work (versus work) contexts and activities.

*Hypothesis 1a*: *Mean-level PA will be higher away from work than at work*.
*Hypothesis 1b*: Mean-level *NA will be higher at work than away from work*.

### Study moderators

While we do offer main effect hypotheses, the mixed evidence above – along with intuition – suggest that there also is considerable variability in work versus non-work affective experiences. Thus, we also examined several moderators of the overall relationships. Specifically, we investigated moderators related to the measurement of affect and related to contextual (i.e., work or non-work) and demographic factors.

One set of moderators we considered involves the measurement of affect. Important to note is that, although we refer to these moderators as reflecting aspects of measurement, the proposed mechanisms underlying their potential effects refer to substantive theoretical phenomena or considerations. For example, for the ESM versus DRM moderator, we propose that the two measurement approaches may capture distinct evaluative processing involved in affective experiences (and in the *reporting* of affective experiences). As such, we regard the moderators (results) as speaking to theoretical questions/issues, rather than just reflecting measurement artifacts.

#### Measurement method: ESM versus DRM

The first possible moderator we consider is whether affect is conceptualized and operationalized using 1) real-time measures capable of capturing moment-to-moment changes/differences (e.g., Experience Sampling Methodology (ESM); or 2) recall/summary measures of affect (i.e., the Day Reconstruction Method; DRM). Kahneman and colleagues developed the DRM as an alternative to ESM [16]. Specifically, the DRM is mean to capture momentary affect in a manner that is less invasive and burdensome for participants than is ESM. In that seminal paper, the authors note strong correlations between data points collected using the two respective approaches [16]. However, the handful of other studies comparing the methods have not confirmed this equivalence [29, 30].

Noting these discrepancies, Diener and Tay called for more systematic investigations comparing these methods [31]. The present meta-analysis offers an opportunity to conduct such a large-scale comparison. More fundamentally, this analysis also can help yield insight into (potentially) distinct experiences of affect (as captured by the respective approaches). Although evidence is mixed, and explanations for potential differences are only speculative at this point [31], we ultimately concluded that there is sufficient evidence to offer a hypothesis here.

In particular, we hypothesize that ESM comparisons of work versus non-work will portray work more favorably compared to DRM comparisons. Answering Diener and Tay’s call to examine whether situational stereotypes may explain divergent findings from the methodologies [31], we propose that this effect results from the differential impact of such stereotypes in the two approaches. Specifically, we suggest that abstractions (i.e., prototypes/stereotypes) of work versus non-work will play a larger role in DRM ratings than in ESM ratings.

We base this argument on Construal Level Theory (CLT) [32, 33]. According to CLT, people may construe of objects and events at differing levels of abstraction ranging from high-level construals (represented in terms of a few abstract features) to low-level construals (represented in terms of concrete and incidental details). The level of abstraction employed in a particular situation depends largely on the *psychological distance* associated with the object or event. In general, CLT proposes that greater psychological distance (whether temporal, spatial, etc.) is associated with higher-level (versus lower-level) construals. According to Trope and Liberman, these higher-level construals not only are more abstract than low-level construals, but they also tend to be simple, decontextualized, and “primary” or “core” in terms of containing the essential/prototypical elements of the object or event [33].

We present a brief illustration of the basic idea of CLT within the context of the current study. In thinking about work *yesterday*, the relative temporal ‘nearness’ should encourage low-level construals which incorporate particular details of one’s work day (e.g., a meeting scheduled with a particular person at a particular time on a particular topic). In contrast, when thinking about work *last month*, or about one’s job more *generally*, the relatively greater psychological/temporal distance should encourage high-level construals which incorporate abstractions and the core/primary elements of one’s work as opposed to very specific tasks and activities associated with a particular workday. Support for CLT comes from studies on a wide-range of topics, employing a wide range of methodologies [33].

Applying CLT here, this theory would seem to imply that, compared to DRM studies, investigations employing real-time methods (e.g., ESM) should encourage low-level construals. This is because the psychological/temporal distance between the participants’ rating is less (i.e., “nearer”) than when participants’ rating is separated in time from the actual experience - as in DRM. Because of the greater temporal distance between the *lived experience* of a work event and the *re-lived experience* of that event in DRM (versus in real-time studies), we expect a corresponding tendency for work to be thought about in more abstract terms here. In turn, we propose that individuals’ held *schemas* about work and working should have a greater influence on reports of affect in DRM studies than in real-time studies.

Thus, to the extent that held schemas about work(ing) tend to skew negative, results of DRM studies likewise should reflect this more negative schema of work. In contrast, because real-time studies should reduce psychological distance and foster lower-level construals, there should be less incorporation of generalized negative schemas about work. While we were not able to locate any theories or direct evidence suggesting that schemas about work(ing) are especially negative, there certainly are bountiful examples from outside the academic literature of work being portrayed as aversive (e.g. portrayals of work in music and in literature). Thus, while there obviously also are counter-examples of work being cast in positive terms, the notion that work more so is conceived in unfavorable terms seems reasonable.

Important to note is that, in making this prediction, we are not implying the superiority of one method over the other one [18]. Rather, we are suggesting the methods capture partly unique affective experiences - affect experienced in the moment (in ESM studies) versus reconstructed affect that incorporates previously held schemas along with reflection about the occurrence in question. As we elaborate upon in the Discussion section, theory and practical study considerations should dictate choice between these approaches.

*H2a*: *The difference between mean PA away from work versus at work (as proposed in*
*Hypothesis 1a) will be smaller in real-time studies than in DRM studies*.
*H2b*: *The difference between mean NA at work versus away from work (as proposed in*
*Hypothesis 1b) will be smaller in real-time studies than in DRM studies*.

#### Operationalization of work: Work as an activity versus as a location

A second potential measurement-related moderator is the operationalization of “work(ing).” In some primary studies included here, work is conceptualized as an activity (i.e., people report that they are/were “working” at a given time). In other studies, work is conceptualized as a domain/location (i.e., people report being at their job location). We see two possibilities regarding the potential effects of this difference in wording.

Borrowing from CLT, one possibility is that reporting about work as an *activity* will result in more contextualized processing (i.e., lower-level construals that are more specific versus abstract generalizations). As such, when reporting about “working” or “doing work,” people’s reported affect should coincide more closely with, or rely on reference to, the actual activities they are completing as opposed to relying on more general abstractions about “work” in a broad, non-task-specific sense. In contrast, when reporting about work as a *location*, there may be a greater disconnect between reported affect and the behaviors or activities in which people are/were engaged. Rather, such reports may allow for a relatively greater influence of schematic thoughts about work versus non-work to color those reports. Again, insofar as there exists a schema of work being aversive, reports of affect between work and non-work should deviate more so in the latter case. As a result of these proposed processes, work should be reported as especially unpleasant relative to non-work when it represents a location.

An alternative notion - and, in turn, prediction - also seems plausible. When people report their affect “at work” (as a location), they may be referencing experiences/activities that are not actually “work” as defined by their job descriptions/roles [34]. Instead, they also may be engaging in and reporting about activities that are perceived as discretionary and favorable, such as socializing with coworkers. As such, affect reports based on work-as-location may lead to relatively higher levels of PA and lower levels of NA as compared to affect reports based on work-as-activity, the latter of which should reflect only task-related activities. Clearly, both of these suggestions are speculative, owing in part to a lack of relevant data regarding how people *think about their work*. Given what we perceive as speculative and equally plausible alternative predictions, we offer competing hypotheses.

*H3a*: *The difference between mean PA away from work versus at work (as proposed in Hypothesis 1a) will be smaller in studies that operationalize work as an activity than in studies that define work as a location*.
*H3b*: *The difference between mean NA at work versus away from work (as proposed in Hypothesis 1b) will be smaller in studies that operationalize work as an activity than in studies that define work as a location*. *VERSUS*
*H3c*: *The difference between mean PA away from work versus at work (as proposed in Hypothesis 1a) will be larger in studies that operationalize work as an activity than in studies that define work as a location*.
*H3d*: *The difference between mean NA at work versus away from work (as proposed in Hypothesis 1b) will be larger in studies that operationalize work as an activity than in studies that define work as a location*.

#### Nature of affect

The primary focus of the meta-analysis is on potential differences in generalized PA and NA between work and non-work. In addition to valence, though, affect varies along a number of other dimensions [35]. Thus, we also examine affect in two additional ways beyond valence. First, applying the conceptualization of affect varying along the dimensions of valence and intensity [36], we explore whether affect intensity moderates the PA and NA findings. In addition to adopting the dimensional view of affect, we also applied the discrete view and examined potential differences between work and non-work affect for specific types of affect (e.g., anger versus anxiety). The decision to consider discrete forms of affect derived from the considerable research showing that even moods and emotions of the same valence and similar intensity are associated with different patterns of cognitive appraisal and action tendencies [37. 38]. While one conceivably could bring theories to bear in making predictions for these two potential moderators, we opted to follow a more exploratory approach here. This was because theoretical predictions are seemingly not obvious for intensity and, while perhaps more possible regarding specific types of affect, were not always testable due to constraints in the number of primary studies assessing particular types.

#### Measurement day

Measurement day refers to whether estimates of non-work affect included only measurements made on workdays (e.g., after coming home from one’s job that day) or on both work and non-work days (e.g., weekends). This distinction is potentially meaningful given some research showing that mood is improved during the weekend relative to weekdays [39]. Ultimately, we determined that existing theory and evidence were not sufficient to allow for a formal hypothesis about this factor; moreover, the information reported in the constituent studies did not always allow for adequate/conclusive coding of whether (or to what extent) individuals’ affect reports were made on the weekend versus during the workweek.

#### Measurement schedule

Measurement schedule refers to the timing of the non-work measurements during the workday. In each study, employees reported their non-work affect before work, after work, or both before and after work. Evidence suggests that there are diurnal rhythms to both positive and negative affect. PA tends to increase during the day, perhaps leveling off in the afternoon [40, 41]. There also is evidence that NA tends to decrease during the day [40], but this pattern is less strong than the one for PA [41]. This is because external events (that can occur at any time) seem to drive negative affect more so than do internal biological systems regulating diurnal rhythms [41]. Still, findings imply that work should be rated as especially aversive when non-work affect (and especially PA) is measured later in the day. We do not offer specific hypotheses for time of day as a potential moderator, in part due to the speculative nature of the above reasoning, and in part owing to the fact that primary studies seldom report specific timings of measurements on the 24-hour clock (e.g., “after work” affect reports could occur at 5 pm or 10 pm, or any other number of times).

#### Job complexity

We also examined potential moderators reflecting the nature of one’s job and one’s non-work life/ demographic factors. The inclusion of these variable derives from the intuitive notion that the degree to which time spent at work is more or less pleasant than time spent elsewhere depends on the nature/characteristics of these two factors/domains.

Turning first to the nature of work, studies document that various job features are associated with salubrious psychological outcomes, including higher PA and lower NA [42]. To capture the many characteristics that potentially lead to differences, we coded for job complexity. Borrowing from other theory and findings, we propose that more complex jobs offer psychological advantages including the opportunity to use and develop a greater amount of knowledge and skills and also more control over one’s work decisions [43]. As such, work-related PA should be relatively higher and work-related NA relatively lower for employees with more complex jobs. This leads to the following predictions.

*H4a*: *The difference between mean PA away from work versus at work (as proposed in*
*Hypothesis 1a) will be smaller for samples with higher job complexity*.
*H4b*: *The difference between mean NA away from work versus at work (as proposed in*
*Hypothesis 1b) will be smaller for samples with higher job complexity*.

#### Educational attainment

We also examined the educational level of the study participants as a potential moderator. We propose that work will be associated with relatively higher PA and lower NA (than non-work) for employees with greater educational attainment for two reasons. First, studies document that education level is positively correlated with various job characteristics [44] that, in turn, predict greater well-being [43]. Also, the higher incomes associated with more educational attainment can help reduce hassles and life events that can occur during non-work time (e.g., not being able to afford healthcare for oneself or one’s family) that negatively impact well-being [16]. Thus, we propose the following hypotheses.

*H5a*: *The difference between mean PA away from work versus at work (as proposed in*
*Hypothesis 1a) will be smaller for samples with higher educational attainment*.
*H5b*: *The difference between mean NA away from work versus at work (as proposed in*
*Hypothesis 1b) will be smaller for samples with higher educational attainment*.

#### Other non-work factors

We also reasoned that one’s circumstances and experiences outside of work could impact affect in that domain (e.g., at home) as well as affect at work (e.g., through family-to-work conflict). Thus, we examined multiple life-circumstance/ demographic variables as potential moderators – namely, the average age of the sample, the proportions of men and women in the sample, and the proportion of the sample that was cohabitating or married (versus living alone).

There is some indirect evidence suggesting directional propositions for these factors. For example, because some research demonstrates a positive relationship between age and job satisfaction [45], positive affect also reasonably should be relatively higher at work for older samples, while negative affect may be lower. Ultimately, though, we concluded that because the supportive evidence relates to attitudinal measures or overall well-being rather than to affect per se, operating in an exploratory manner here was appropriate.

## Materials and methods

### Literature search

We conducted a broad search using the Science Direct databases (PsycInfo, PsycArticles) with the following search terms: “experience sampling,” “event* sampling,” “ESM,” “day reconstruction,” “DRM,” “ecological momentary,” “EMA,” “ambulatory assessment,” or “daily diary.” We also searched the Web of Science database using our initial search terms in combination with the following terms: “mood,” “emotion*,” affect,” or “well*being.” We did not specify start dates for any of the searches. The Science Direct databases contain comprehensive records dating back to the late 1800’s. The Web of Science database contains records dating back to 1900. The searches concluded in October, 2015. Also in October 2015, we conducted a targeted Google Scholar search for any additional datasets published by authors of existing usable datasets we previously had located.

### Criteria for inclusion

Our inclusion criteria were the following: (1) sample of employed adults, excluding undergraduate student samples and clinical treatment groups, (2) use of real-time or short-term retrospective methods to measure affect during the current or previous day, (3) study included at least one emotion that could be categorized as either PA or NA, or included a measure of PA and/or NA, and (4) study design included measurement of affect during work and during non-work on the same day(s). Regarding the fourth requirement, we did include studies that measured affect on days when participants were not working (e.g., weekends) provided that both work and non-work also were assessed on the same day other day(s).

We identified approximately 350 studies that met these criteria. However, the majority of these studies did not report descriptive statistics separately for work and non-work affect as was required for the analyses. In those cases, we contacted the corresponding author via email (and, with a second message in cases of non-response) to request the necessary information. For approximately 10% of our requests, authors provided us with necessary information – either statistics or raw data – for the analyses.

For nineteen papers and two publically available data sets [46, 47], we computed the required statistics directly from raw data. We did so using the following method. First, we calculated average PA and NA associated with work and non-work, respectively, for each day for each participant. Next, we calculated person averages by aggregating across days for each participant. Finally, from these person averages we calculated sample averages and standard deviations for PA and NA that were in turn used for computing ESs.

**Fig 1:**
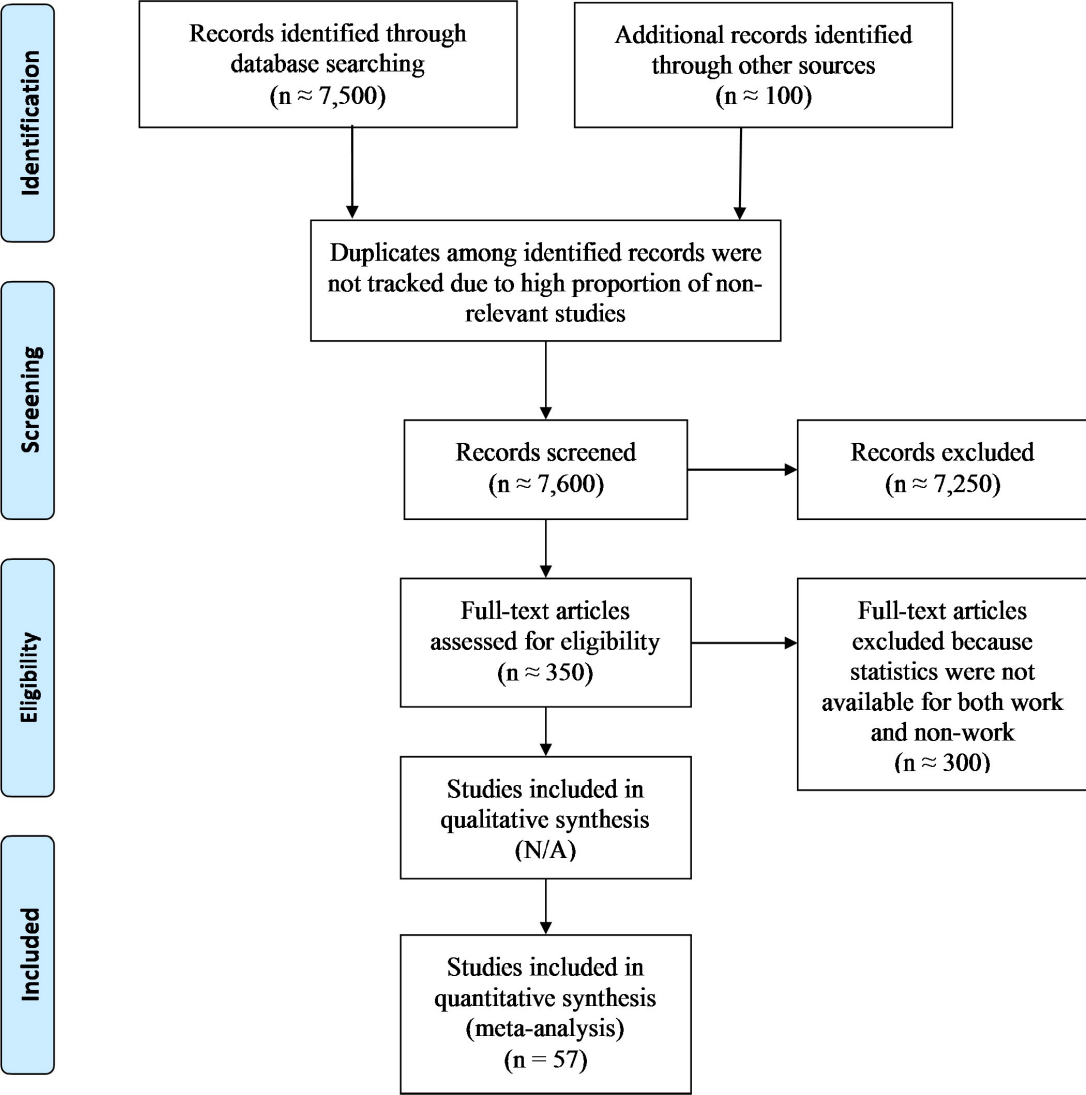
PRISMA flow diagram for studies included in and excluded from the meta-analysis.

The final database included 57 published papers or dissertations and the two publicly available datasets for which we were able to calculate relevant effects. Corresponding references are provided in the S1 Appendix. Fig 1 is a flow diagram depicting the inclusion process for the final 57 studies. For PA, these studies provided 51 effect sizes based on a total sample of 37,472 participants and an estimated 1.6 million measurement occasions. For NA, these studies provided 53 effect sizes based on a total sample of 16,970 participants and an estimated 300,000 measurement occasions. Table 1 provides additional sample characteristics. S1 Table provides the full details from the primary studies.

**Table 1:** Summary of included studies.

Study Characteristics	Positive Affect		Negative Affect	
	*K*	*N*	*K*	*N*
Measurement method				
Real-time	37	24,876	39	4,473
DRM	8	12,011	8	12,005
Operationalization of work				
Location	30	3,282	37	4,005
Activity	18	33,857	13	12,635
Measurement day				
Workdays only	34	7,763	37	8,050
Workdays and weekends	17	29,709	16	8,920
Measurement schedule away from work				
Before work only	1	68	2	103
After work only	11	927	11	924
Before and after work	36	36,343	37	15,809
Total	51	37,472	53	16,970

### Coding of studies and operationalization of variables

All four authors were involved in coding studies. First, all authors coded a common subset of studies to ensure consistent interpretations and to allow for refinement of decision rules. Subsequently, two authors (the first author and one of the three other authors) coded each of the remaining studies.

Agreement between coders was generally strong. Most discrepancies reflected typos or mistakes in extracting information from papers or data sets. For more substantive discrepancies, the authors reached agreement through discussion of the study parameters and inclusion criteria. We calculated agreement between each pair of coders prior to resolution of the discrepancies [48]. Initial agreement was 77.9% for affect categorization (i.e., whether an emotion was appropriately categorized as PA or NA) and averaged 80.2% for moderator variables (range across moderators = 72.1 – 88.5%).

For studies that reported discrete affect variables (e.g., happy, anxious), we categorized those variables as an aspect of either PA or NA based on the PANAS-X manual [49]. A discrete emotion was categorized as PA if it was consistent with either the General Dimension Scale for PA or any of the Basic Positive Emotion Scales (i.e., Joviality, Self-Assurance, Attentiveness). Similarly, a discrete emotion was categorized as NA if it was consistent with the General Dimension Scale for NA or any of the Basic Negative Emotion Scales (i.e., Fear, Hostility, Guilt, Sadness). We also categorized stress as NA if the items composing the measure were consistent with Watson and Clark [49] or when the emotion was simply stated (e.g., “I feel stressed”). In addition to recording information necessary for calculating main effects, we also coded the following study-level variables as potential moderators: measurement method, operationalization of work, measurement day, measurement schedule, affect intensity, job complexity, educational attainment, age, gender, and relationship status.

We first describe the operationalizations of potential moderators related to the measurement of affect and then those related to contextual work and demographic factors. *Measurement method* refers to a binary variable denoting whether the study used 1) *real-time* measures of affect (e.g., ESM, ecological momentary assessment (EMA); or 2) the DRM. *Operationalization of work* is a binary variable referring to whether a given study defined and measured “work” in terms of 1) an *activity* (i.e., “working” or “doing” work) versus; 2) as a *location* (i.e., “at the office/workplace”). *Measurement day* is a binary variable created to distinguish between studies in which estimates of non-work affect included measurements on 1) *workdays only* (i.e., excluded any measurements from weekends and other non-working days) or; on 2) *both* work and non-work days (i.e., *workdays and weekends*). *Measurement schedule* pertains to the timing of the non-work measurements: 1) *before work only*; 2) *after work only*; or 3) *before and after work*. Last, *intensity of affect* refers to the overall level of activation or arousal associated with a measure of affect. For each study, we calculated the intensity of PA and/or NA as the average arousal of the measure’s items, utilizing arousal established norms [50].

Turning to the work and non-work factors, we followed others [51] in operationalizing *job complexity* using the O*NET “Job Zones” [52]. A Job Zone is a group of occupations that are similar in how much overall experience, education, and on-the-job training people need to be able to do the work. There are five Job Zones, ranging from 1 (little or no preparation) to 5 (extensive preparation). Since most of the samples were mixed in terms of jobs, we calculated weighted averages when studies reported the information allowing us to do so (e.g., [% Zone 1]*[1] + [% Zone 2]*[2] + [% Zone 3]*[3] + [% Zone 4]*[4] + [% Zone 5]*[5]). However, the majority of studies in our sample did not provide enough information to calculate this composite score. Therefore, we contacted study authors and asked them to report the percentage of the sample that corresponded to each of the job zones. Some authors did send us raw data consisting of participants’ job titles. In these cases, two coders (the first author and one additional author) coded each of job titles using O*NET. Agreement between the composite scores created by the two coders was generally strong and did not lead to different analysis conclusions. Therefore, we report results for job complexity based on the average of the two coders’ ratings. In total, we were able to make assessments for 16 studies that covered a range of overall job complexity (*M* = 3.39; *SD* = 0.93).

Regarding the remaining potential moderators, we coded *educational attainment* as the percent of the sample with a bachelor’s degree or higher. *Age* refers to the average age of participants in the sample, and *gender* is the percent of the sample that was female. Last, *relationship status* was coded as the percent of the sample that was married or cohabitating.

### Effect sizes

At the individual study level, effect sizes (ES) were calculated as the mean standardized difference between work PA/NA and non-work PA/NA, (*M*_work_ – *M*_non-work_)/*SD*_pooled_. Positive ESs indicated that affect (either PA or NA) was higher for work than non-work. A number of studies reported multiple estimates for either work or non-work affect. In these cases, we typically calculated simple averages within each domain before computing an ES. For example, if two time points were associated with work, one in the morning and one in the afternoon, we averaged the two time points for the overall analyses.

However, some studies reported additional information that allowed us to calculate weighted averages. Real-time studies sometimes reported N (i.e., the number of surveys completed) separately for each time point. For example, an article could mention that there were 1,000 assessments of affect “after work” but only 900 assessments of affect “before work” on a given day. In instances like this, we calculated a weighted average of non-work affect. In addition, DRM studies sometimes reported the average amount of time participants spent in the various activities for which they made affect ratings. In these cases, we time-weighted the activity estimates when computing overall averages for work and non-work affect.

This aggregation was necessary in order to maintain independent data points within the analysis. Thus, we effectively reduced the complexity of the data to a standardized mean gain where the same variable is measured on two occasions (i.e., work and non-work) for each sample [53]. For this within-subjects effect, *SD*_pooled_ is based upon the between-person standard deviations for both work and non-work. When studies did not report means and/or standard deviations, we attempted to calculate ESs using other statistics [53]. For studies that reported F-statistics for one-way ANOVAs of two independent group means, we calculated ES using the following formula where *n* = sample size, ES = (2*F*/*n*)^0.5^ [53]. In cases where t-statistics were reported, we used either the independent t-test conversion formula, ES = 2*t* / (2*n*)^0.5^ [53] or the repeated measures formula, ES = *t*[2(1-*r*)/*n*]^0.5^ [54] as appropriate. When only regression coefficients were reported, we made the following calculation, ES = *B* / *SD*_pooled_ [53].

Several studies reported multiple discrete emotions that were aspects of either PA or NA. In these cases, we combined these discrete emotions into a respective PA or NA composite for the comparisons involving generalized PA and NA. For example, estimates of happy, confident, and cheerful in one study [55] were combined to create an average for PA. Each effect size was subsequently corrected for unreliability [56] and then weighted by its inverse variance using the following formula for repeated measures, 2n/[4(1-*r*) + ES^2^] [53]. In this formula, *r* is the person-level correlation between PA/NA associated with work and PA/NA associated with non-work. When reliability (alpha) or *r* was unavailable for a study, we used the average from other studies in the sample [56]. For studies with single-item measures, we assumed an estimated reliability of .70 [57]. Main effects analyses conducted both with and without corrections for unreliability revealed consistency in the substantive findings; the results reported herein include both observed and corrected effects.

Overall ESs and associated heterogeneity were computed using random-effects models. Where the *Q* statistic for a main effect suggested variation in true ESs across studies, we also tested for the presence of moderators using mixed-effects modeling. Compared to fixed effects meta-analysis, the random effects models provided more realistic confidence intervals and allows for making make inferences extending beyond the studies in the present analysis [58]. For each model, we identified influential outliers using features of the Metafor package for R (Version 1.9-8) and procedures outlined here [59, 60]. An individual study ES qualified as an outlier if its studentized deleted residual exceeded ±1.96. Outlier ESs were then further evaluated using the *influence* function in R to examine the DFFITS value, Cook’s distance, leverage (hat value), and DFBETAS [59]. When ESs were identified as both outliers and influential cases, we ran analyses both with and without those cases. For most models, we retained the influential outlier studies since their inclusion/exclusion did not impact conclusions. However, based on these criteria, a total of three studies were removed from the main effects models. One effect size was removed from the NA analyses [61]; and two were removed from the PA analyses [13, 62].

Outlier diagnostics were run separately for the moderator analyses. In the univariate NA models, one of the studies noted above was removed from all moderator analyses for which it qualified [61]. One study was removed from the measurement day model for the univariate NA model [63]. Three of the NA models excluded two additional studies: the operationalization of work model [63, 64], the measurement schedule model [64, 65], and the gender model [66, 67].

In the univariate PA models, [13] was again removed from all analyses for which it included moderator data. Two of the PA models excluded one additional study: the measurement method model [27], and the age model [62]. The PA model for measurement schedule excluded two additional studies [62, 68] as did the model for measurement breadth [68, 69]. Finally, the PA model for operationalization of work excluded three additional studies [23, 27, 62].

In the multivariate moderator analyses, one study was removed from the NA model [61]. No outliers were removed from the multivariate model for PA [61]. All statistical analyses were conducted using the Metafor meta-analysis package for R (Version 1.9-8) [59, 60], except where otherwise indicated.

## Results

### Main effects

One study was identified as an influential outlier for NA and two studies were identified as influential outliers for PA. However, there were no substantive reasons to exclude the studies so they were retained for the analyses [70]. Moreover, the conclusions for the main effects remained the same, and the meta-analytic effect sizes changed only slightly when these effects were excluded (observed *d* for PA changed by .05 and for NA changed by .03).

An additional consideration was that several of the studies have substantially larger sample sizes compared to the remainder of the studies. In particular, one study had an especially large sample (*N* = 20,946) [28] and three other studies had sample sizes exceeding 1,000 participants (total *N* = 10,531). In contrast, the remaining 56 studies had relatively more modest sample sizes (average *N* = 122; total *N* = 6,808). Since the four larger samples may be more influential and/or might differ systematically compared to the others (e.g., perhaps having more nationally representative samples), we conducted the analyses both with and without those studies included. Once again, the conclusions for the main effects remained unaltered, and the impact of the large studies on the meta-analytic effect sizes was small (observed *d* for PA changed by .04 and for NA changed by .01). Given this lack of impact, we retained the large studies in the analyses.

**Fig 2:**
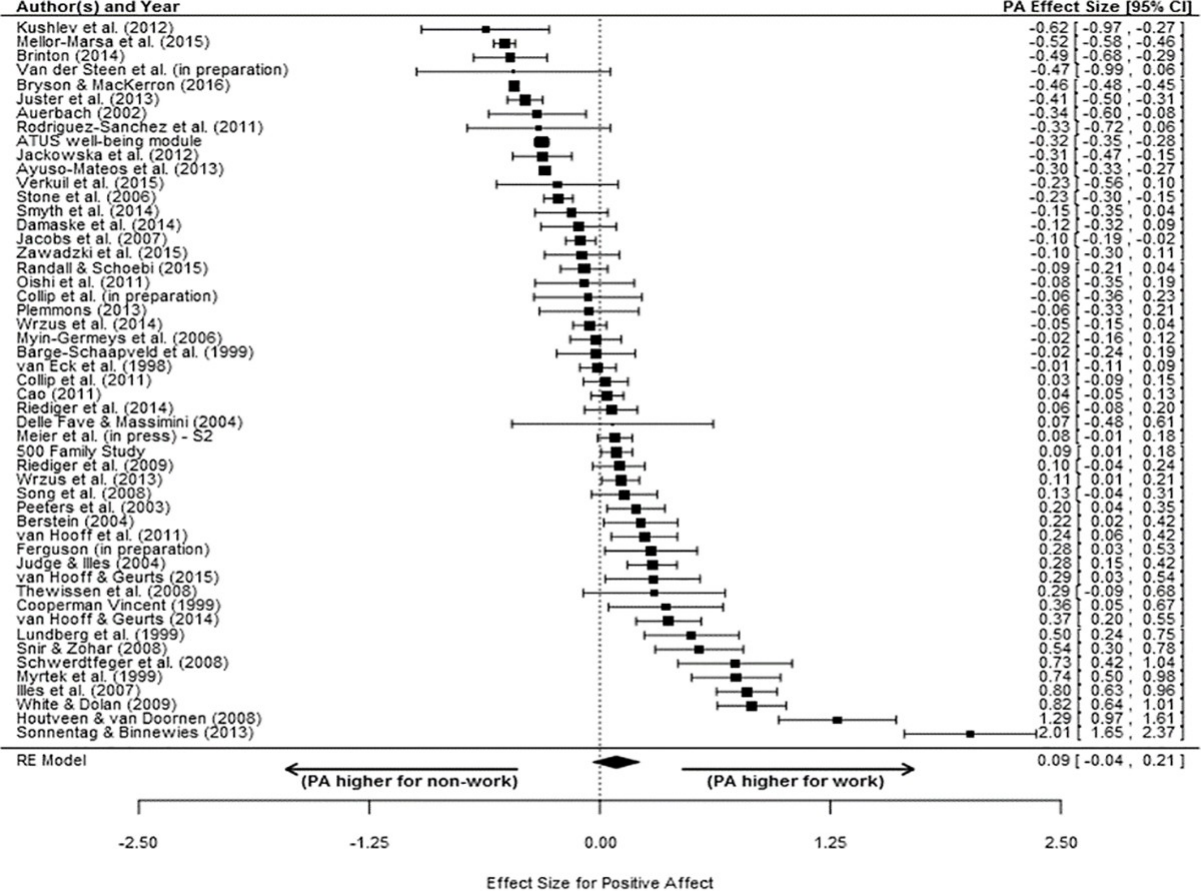
Effect sizes (ESs) comparing work positive affect to non-work positive affect. For each independent sample included in the meta-analysis, a corrected ES (square) and the associated 95% confidence interval (CI; line) is shown. On the bottom, the diamond shows the meta-analytically inverse variance weighted mean ES. The values associated with the ESs and CIs are located in the right column. Positive values for ES indicate greater positive affect during work versus non-work.

**Fig 3:**
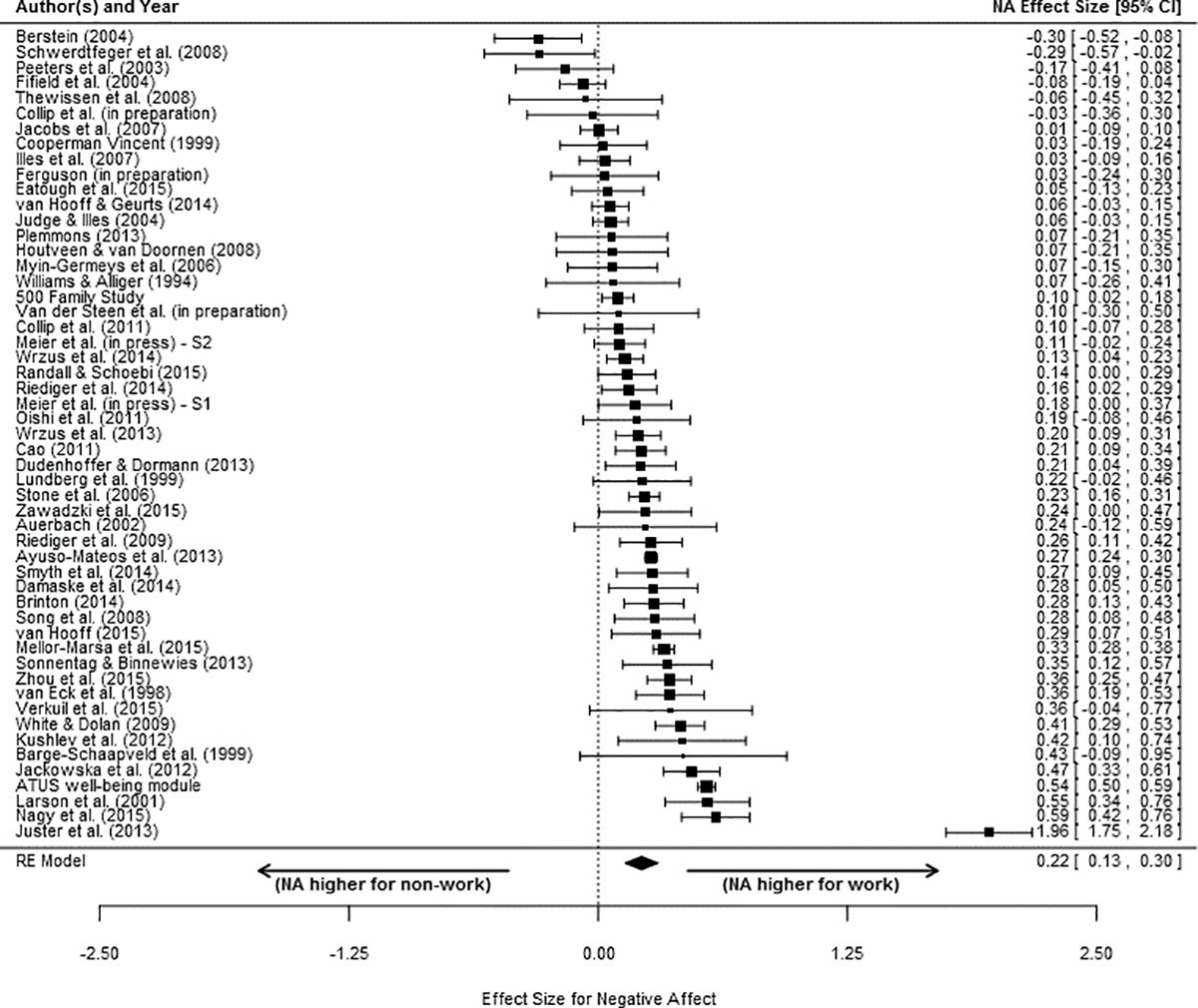
Effect sizes (ESs) comparing work negative affect to non-work negative affect. For each independent sample included in the meta-analysis, a corrected ES (square) and the associated 95% confidence interval (CI; line) is shown. On the bottom, the diamond shows the meta-analytically inverse variance weighted mean ES. The values associated with the ESs and CIs are located in the right column. Positive values for ES indicate greater negative affect during work versus non-work.

The individual study ESs and the meta-analytic average ES for PA and NA are shown in Figs 2 and 3, respectively. The inverse variance weighted effect for PA was positive but not statistically significant (*d* = .07; δ = .09, 95% CI [-.04, .21], *p* =.160). In contrast, the inverse variance weighted effect for NA was positive and statistically significant (*d* = .18; δ = .22, 95% CI [.13, .30], *p* < .001). These results indicate that, on average, individuals do *not* report feeling more positive affect when away from work/not working than when at work/working. In contrast, reports of negative affect were significantly higher for work versus for non-work - although the magnitude of the difference was small. Notably, these conclusions were robust and not dependent on the exclusion of any individual study. Thus, Hypothesis 1a was not supported, whereas Hypothesis 1b was supported. In each case, heterogeneity analysis suggested a high degree of variability in individual study effect sizes after accounting for sampling error (*Q*_PA_ = 2140.42, *p* < .001; *Q*_NA_ = 674.39, *p* < .001). As such, we examined potential moderators.

### Moderators based on the measurement of affect

The results of the univariate moderator analyses for PA and NA are reported in Tables 2 and 3, respectively. In Hypothesis 2a and 2b, we predicted mean differences in affect as a function of “measurement method” – that is, whether the study used a real-time data collection method (e.g., ESM) or the day reconstruction method (DRM). For the univariate moderator analyses associated with these two hypotheses, outlier diagnostics identified one influential outlier study for PA and one (other) influential outlier study for NA. Although the magnitude of the meta-analytic effects was not substantially altered by the outliers, their exclusion changed the results of the moderator analyses from nonsignificant to statistically significant. The following results exclude the outlier studies and thus should be interpreted with a degree of caution. As seen in Table 2, measurement method was a significant moderator for PA (*Q*_between-groups_ = 10.84, *p* = .001). Work PA was slightly *higher* than non-work PA in real-time studies (δ = .09, 95% CI [-.01, .19], *p* = .078). In contrast, work PA was significantly *lower* than non-work PA in DRM studies (δ = -.33, 95% CI [-.56, -.10], *p* = .004). This finding supported H2a. The result for NA also was consistent with the relevant prediction in H2b (*Q*_between-groups_ = 11.80, *p* < .001). Although work NA was higher than non-work NA for both types of studies, the difference was stronger in DRM studies (δ = .36, 95% CI [.26, .46], *p* < .001) than in ESM studies (δ = .16, 95% CI [.10, .21], *p* < .001). These findings support the idea that work is regarded as relatively more pleasant and less aversive when reports are made in real-time versus retrospectively.

**Table 2:** Univariate moderator analyses for positive affect: Work versus non-work.

Moderator	*k*	*d*	*δ*	*SE*	95% CI	*p*	Moderator *Q*	*p*
Measurement method								
Real-time	37	0.08	0.09	0.05	-.01, .19	0.078	10.84	0.001
DRM	7	-0.28	-0.33	0.11	-.56, -.10	0.004		
Operationalization of work								
Location	30	0.12	0.13	0.08	-.03, .29	0.123	0.22	>.250
Activity	18	0.05	0.06	0.11	-.14, .28	0.549		
Measurement day								
Workdays only	34	0.14	0.16	0.07	.01, .31	0.027	3.19	0.074
Workdays & weekends	17	-0.05	-0.06	0.1	-.27, .14	>.250		
Measurement schedule^a^								
After work only	11	0.39	0.43	0.12	.17, .68	<.001	8.91	0.002
Before and after work	36	-0.01	-0.009	0.07	-.14, .13	>.250		
	*k*	*B*	B*	*SE*	95% CI	*p*		
Affect intensity	51	0.037	0.037	0.11	-.18, .25	>.250		
Job complexity	12	0.033	0.034	0.17	-.31, .38	>.250		
Average age	37	0.021	0.024	0.01	.004, .044	0.015		
% female	39	0.002	0.002	0.002	-.003, .008	>.250		
Educational attainment	18	0.004	0.004	0.002	-.000, .009	0.069		
% married/ cohabitating	25	-0.002	-0.002	0.004	-.011, .007	>.250		

Note: The first set of *p* values indicates whether the predicted estimate differs significantly from zero within moderator level. The second set of *p* values indicates whether the moderator accounts for a significant amount of heterogeneity (Q) in the mixed effects model. *d* = standardized mean difference; *δ* = corrected standardized mean difference; *B* = meta-regression coefficient; B* = corrected meta-regression coefficient.

^a^“Before work only” measurement schedule for non-work affect was excluded from the analysis because it was utilized in only one study.

**Table 3:** Univariate moderator analyses for negative affect: Work versus non-work.

Moderator	*k*	*d*	*δ*	*SE*	95% CI	*p*	Moderator *Q*	*p*
Measurement method								
Real-time	38	0.14	0.16	0.02	.10, .21	<.001	11.80	<.001
DRM	8	0.30	0.36	0.05	.25, .46	<.001		
Operationalization of work								
Location	37	0.19	0.21	0.05	.11, .31	<.001	0.04	>.250
Activity	13	0.16	0.19	0.08	.02, .36	0.025		
Measurement day								
Workdays only	37	0.16	0.18	0.05	.08, .28	<.001	1.22	>.250
Workdays & weekends	16	0.25	0.28	0.07	.13, .43	<.001		
Measurement schedule								
Before work only	2	0.13	0.13	0.20	-.26, .54	>.250	1.79	>.250
After work only	11	0.12	0.13	0.09	-.04, .31	0.128		
Before and after work	37	0.23	0.26	0.05	.17, .36	<.001		
	*k*	*B*	B*	*SE*	95% CI	*p*		
Affect intensity	52	0.029	0.012	0.12	-.22, .25	>.250		
Job complexity	14	0.020	0.022	0.06	-.10, .14	>.250		
Average age	42	-0.001	-0.000	0.010	-.021, .019	>.250		
% female	42	-0.000	-0.000	0.002	-.005, .003	>.250		
Educational attainment	21	-0.001	-0.001	0.002	-.006, .003	>.250		
% married/ cohabitating	22	-0.003	-0.004	0.004	-.012, .004	>.250		

Note: The first set of *p* values indicates whether the predicted estimate differs significantly from zero within moderator level. The second set of *p* values indicates whether the moderator accounts for a significant amount of heterogeneity (Q) in the mixed effects model. *d* = standardized mean difference; *δ* = corrected standardized mean difference; *B* = meta-regression coefficient; B* = corrected meta-regression coefficient.

In Hypotheses 3a-3d, we offered competing predictions in regard to whether work was operationalized as a location versus an activity. Findings showed that this distinction was not significant for PA (*Q*_between-groups_ = .22, *p* = >.25) or NA (*Q*_between-groups_ = .04, *p* > .25). Thus, neither set of hypotheses was supported. The next moderators of interest pertained to the nature of affect. First, we investigated whether affect intensity (i.e., activation or arousal) impacted the difference in work- versus non-work PA and NA. Meta-regression results revealed that there was no effect of intensity of affect for either PA (B* = .03, *p* = >.25) or NA (B* = .01, *p* = >.25). Second, we also examined potential differences in work versus non-work experiences for specific types of affect. As seen in Table 4, we computed the meta-analytic effects for 23 discrete emotions where effect sizes could be calculated for 3 or more studies. Regarding positively valenced discrete emotions, alert (*d* = .37, *p* = .003), cheerful (*d* = .02, *p* = .025), and interested (*d* = .26, *p* = <.001) were higher for work than for non-work, whereas enjoying (*d* = -.31, *p* = <.001) and happy (*d* = -.19, *p* = .005) were lower for work than for non-work. Regarding negatively valenced discrete emotions, ten emotions were higher for work than for non-work: angry (*d* = .14, *p* = .008), depressed/blue (*d* = .05, *p* = .017), frustrated/annoyed (*d* = .39, *p* = <.001), insecure (*d* = .13, *p* = .008), irritated (*d* = .08, *p* = .001), nervous (*d* = .22, *p* = <.001), sad (*d* = .11, *p* = <.001), stress (*d* = .35, *p* = <.001), tense (*d* = .23, *p* = .013), and worried/anxious (*d* = .16, *p* = .007). Of note, these effect sizes generally were modest in magnitude as well (perhaps with the exceptions of frustrated/annoyed and stress). The effects for the other discrete emotions were not statistically significant. We expand on these results below.

**Table 4:** Discrete emotions during work and non-work.

Discrete Emotion	*k*	*N*	*d*	*SE*	95% CI	*p*
Agitated	3	96	0.16	0.19	-.20, .54	>.250
Alert	3	182	0.37	0.12	.12, .62	0.003
Angry	10	1435	0.14	0.05	.03, .25	0.008
Cheerful	10	1464	0.04	0.01	.00, .07	0.025
Depressed/Blue	11	8894	0.05	0.02	.00, .09	0.017
Down/Downhearted	9	940	-0.01	0.02	-.06, .02	>.250
Enjoying	5	8260	-0.31	0.05	-.42, -.20	<.001
Enthusiastic	4	198	0.15	0.09	-.03, .34	0.117
Excited/Energetic	4	293	0.24	0.14	-.03, .53	0.087
Frustrated/Annoyed	5	1850	0.39	0.07	.24, .54	<.001
Guilty	8	724	-0.01	0.06	-.15, .11	>.250
Happy	16	26137	-0.19	0.07	-.33, -.05	0.005
Insecure	7	691	0.13	0.04	.03, .22	0.008
Interested	3	279	0.26	0.04	.17, .35	<.001
Irritated	10	1492	0.08	0.02	.03, .13	0.001
Lonely	9	1297	-0.02	0.05	-.12, .07	>.250
Nervous	6	1323	0.22	0.05	.12, .32	<.001
Sad	5	3735	0.11	0.01	.08, .14	<.001
Satisfied	8	724	-0.08	0.05	-.18, .00	0.076
Stress	10	4611	0.35	0.07	.21, .49	<.001
Tense	4	219	0.23	0.09	.04, .41	0.013
Vitality	3	191	0.34	0.39	-.42, 1.10	>.250
Worried/Anxious	18	9964	0.16	0.06	.04, .28	0.007

Note: *d* = standardized mean difference. The corrected standardized mean difference is not reported since the majority of measures consisted of single items.

Finally, we also investigated when measurements were made. For “measurement day”, results showed that whether or not the non-work measurements also occurred on weekends did not significantly moderate the results for PA (*Q*_between-groups_ = 3.19, *p* = .074) or NA (*Q*_between-groups_ = 1.22, *p* > .25). Of note, though, the result for PA approached the *p* < .05 significance level, with work being rated as relatively more pleasant when non-work affect was only assessed on weekdays rather than on both weekdays and weekends. Additionally, the timing of the non-work measurements on workdays (i.e., measurement schedule) was a significant moderator for PA (*Q*_between-groups_ = 8.91, *p* <.001). For studies with non-work measurements occurring both before and after work, there was no difference in PA between work and non-work (δ = -.01, 95% CI [-.14, .13], *p* >.25), whereas for studies with non-work measurements occurring only after work, PA was greater during work (δ = .43, 95% CI [.17, .68], *p* = < .001). There was no difference between the measurement schedules for NA (*Q*_between-groups_ = 1.79, *p* = >.25).

### Moderators based on work and non-work factors

Turning to work and non-work factors (i.e., contextual and demographic factors), we first examined the potential influence of job complexity. Contrary to Hypotheses 4a and 4b, job complexity did not moderate the effect for either PA (B* = .03, *p* = >.25) or NA (B* = .02, *p* = >.25). Also, with regard to educational attainment, neither Hypothesis 5a nor Hypothesis 5b was supported. Of note, though, the effect for PA was significant at *p* = .069 (B* = .004) – lending support to the idea that individuals with more education experience relatively greater positive affect at work compared to away from work than do those with lower educational attainment.

With regard to the demographic factors we assessed in an exploratory manner, the average age of the sample was a significant moderator for PA (B* = .02, *p* = .015), but this significant effect only emerged once two influential outlier studies were removed from the analysis. Average age was not a significant moderator for NA (B* = -.000, *p* > .25). Tentatively, older workers reported experiencing relatively higher PA, but not less NA, for work versus non-work than did younger employees. In contrast, neither the gender composition of the sample nor the proportion of the sample cohabitating/married (versus living alone) was a significant moderator for PA or NA (see results in Tables 2 and 3).

### Multivariate moderator analyses

In order to assess potential confounding among the moderators, we conducted multiple regression analysis including the significant predictors. For PA, there were three significant moderators. As shown in Table 5, all three of the factors - measurement method (B* = -.31, *p* = .013), measurement schedule (B* = -.34, *p* = <.001), and average age (B* = .02, *p* = .009) - still contributed to effect size variability in the multivariate model. However, the multivariate finding for age should be interpreted with caution since the significant effect only emerged after the removal of a single influential outlier study. For NA, multiple regression analysis was not required because there was only one significant univariate moderator (measurement method).

**Table 5:** Multivariate model for moderators of positive affect: Work versus non-work.

Moderator	*B*	B*	*SE*	*p*
Measurement method	-0.27	-0.31	0.12	0.013
Measurement schedule	-0.31	-0.34	0.09	<.001
Average age	0.02	0.023	0.009	0.009
			*Q*	*p*
Model			26.60	<.001
Residual			484.77	<.001

Note: *k* = 29. *B* = meta-regression coefficient; B* = corrected meta-regression coefficient.

### Supplemental analysis

As stated previously, the weighting formula for the ESs required the person-level correlation between work PA and non-work PA and between work NA and non-work NA. Thus the data collected for our primary meta-analysis can also be used to calculate meta-analytic estimates of the correlations among these four variables - work PA, work NA, non-work PA, and non-work NA - for a subset of 30 studies in our sample that assessed both positive affect and negative affect. These correlations were calculated using random-effects modeling within the RBNL meta-analysis program [71] and are provided in Table 6. The results show that work and non-work PA were strongly correlated as were work and non-work NA (*ρ*s = .90, .95). In addition, PA was moderately and negatively correlated with NA within each domain (*ρ*s = -.40, -.47) whereas these relationships were somewhat less strong across domains (*ρ*s = -.26, -.30).

**Table 6:** Meta-Analytic correlations for PA and NA during work and non-work.

							80% CV		95% CI	
Constructs	*k*	*N*	$\bar{r}$	P	*SD*_ρ_	% Var.	L	U	L	U
PAwork - NAwork	30	6,493	-0.32	-0.47	0.17	14.37	-0.69	-0.25	-0.54	-0.40
PAnonwork - NAnonwork	30	6,493	-0.28	-0.40	0.15	20.40	-0.59	-0.21	-0.46	-0.35
PAwork - NAnonwork	30	6,493	-0.20	-0.30	0.13	28.54	-0.46	-0.14	-0.35	-0.24
PAnonwork - NAwork	30	6,493	-0.17	-0.26	0.15	22.67	-0.45	-0.07	-0.32	-0.20
PAwork - PAnonwork	30	6,493	0.63	0.90	0.12	10.01	0.74	1.00	0.85	0.94
NAwork - NAnonwork	30	6,493	0.66	0.95	0.12	9.38	0.80	1.00	0.91	1.00

Note: $\bar{r}$ = sample-weighted mean correlation; ρ = estimate of population correlation corrected for unreliability; % Var. = percentage of variance explained by artifacts. Upper limits of 80% credibility value that exceeded 1 are reported as 1

## Discussion

Classical economic theory suggests that work is a relatively distasteful experience. Indeed, Adam Smith, the father of modern economics, described work as “toil and trouble” requiring the sacrifice of one’s “ease, liberty, happiness” [72]. Some prominent modern-day studies support this view, showing work to be among the least pleasant experiences of daily life 16, 28].

However, the current meta-analytic results partly refute this conclusion and instead show that affect at work is nearly on par with affect during the rest of daily life (non-work). Specifically, we found no significant difference between work and non-work positive affect. While work-related negative affect was higher than non-work negative affect, the magnitude of this difference was small, with an effect size of .22 *SD*. To put this effect size in context, other findings suggest that there is as much, if not more, variability in NA experienced during different leisure activities. For example, differences in NA between watching TV and playing board games and between socializing with friends and playing sports are very similar (*d*_[television *–* games]_ = .19 *SD*; *d*_[socializing *–* sports]_ = .20 *SD*) – computed here from the sample of full-time employees in the American Time Use data [47]. This said, the results also showed that several discrete negative emotions were higher for work than non-work.

Below, we expand on this and the other findings. In this discussion, we also note potential directions for future research to more directly test the ideas implied by these results. Following that, we describe potential boundary conditions and limitations of this meta-analysis.

### Interpreting the current findings

Given the variability among the primary study effect sizes, and the presence of at least some significant moderators, a reasonable question is whether the main effects are meaningful. In statistical parlance, “should one interpret the main effects given the significant interactions?” Based on the pattern of findings, we adopt a middle ground position here. On one hand, the heterogeneity in effect sizes and the presence of moderators indicate that the difference between work and non-work affect certainly depends on various factors. On the other hand, several findings *collectively* suggest that the main effect results also convey important information. These four findings are a) the non-significant main effect for PA, b) that work PA is actually slightly higher than non-work PA in studies using real-time methods, c) the relatively small overall effect size for NA, and d) the non-significant results for some of the more intuitive moderators (e.g., job complexity, whether or not weekend affect was taken into account).

With regard to what may explain these findings – especially vis-à-vis contradictory notions or understandings about the emotional experience of work – the moderator results offer some possible insights. Turning first to the method of measurement, work was rated as more pleasant and less unpleasant when studies used real-time methods versus the DRM. This difference was particularly notable for PA; real-time assessments suggested that work PA was slightly higher whereas DRM studies suggested that people felt better in non-work scenarios. Although the impact of work versus non-work on PA was not dramatic for either measurement method, the shifting direction of the effect supports the idea that DRM and ESM capture somewhat different realities [73] as well as the idea that work is associated with different realities. This notion is consistent with Kahneman and Riis’s seminal piece on (measuring) psychological well-being, titled, “Living, and thinking about it: two perspectives on life” - a main thesis of which is that feeling in the moment and subsequent reflection about that moment reflect different realities [74]. Applying this idea (and findings, [75]) to the current results, neither the DRM nor real-time results is wrong. Both are valid reflections of people’s assessments of work; they simply reflect different working realities.

The DRM versus ESM results also seem to have implications with regard to researchers choosing one approach or the other for primary studies. While more work needs to be done, the current results paired with some other findings [18, 73] suggest that real-time methods may be optimal when one is interested in emotion associated with relatively short events or activities (e.g., meetings, common work tasks) and, in particular, on the temporal unfolding of affect over the course of such events or activities. DRM may be less useful for these purposes because DRM produces only a single set of affect ratings if the event occurs during one period on the DRM form [73]. The issue of how people aggregate disparate levels or types of affect over a given experience remains unresolved. Beyond that consideration, though, DRM does not capture the diversity or change in affective experience over a given event.

In contrast, DRM appears more useful for measuring schematic representations of events (and the affect associated with them). To be clear, DRM ratings do capture within-day variability in affect [40]. Thus, they clearly not only capture schemas or stereotypes about different contexts. But, the current findings provide further, albeit indirect, evidence suggesting that DRM ratings are more a function of such schemas than are ESM ratings. Again, choice among these methods, and also regarding the use of overall, one-time measures (or the decision to use multiple methods) should reflect theoretical and practical considerations [73].

This idea that people’s recalled summary reports about work are less pleasant than their in-the-moment experiences of it also warrants separate mention. We are not aware of studies trying to assess schemas or stereotypes about the experience of working. But, assuming for the moment that future studies addressing this phenomenon are supportive, a reasonable query is, “now what?” Practically speaking, there is a question of whether organizations might develop interventions to reveal this disconnect and the notion that we sometimes “enjoy work more than we think we do.” We are not advocating such an intervention at this point but simply raise it as a possibility for future consideration and research.

The potential for this disconnect between experienced versus recalled affect also suggests value in trying to determine the features of work whose affective utility individuals may underestimate. Large-scale survey studies indicate that people believe extrinsic factors (e.g., pay, prestige, benefits) are especially strong contributors to job satisfaction [76]. Correlational studies among various factors and job satisfaction ratings, however, do not support the centrality of these extrinsic features for satisfaction. Rather, aspects of the job and the social environment consistently emerge as stronger predictors [77]. The current findings may suggest that there is a tendency to focus on extrinsic factors when reporting job attitudes, perhaps more so than when reporting on momentary affect - when job features and social phenomena are more proximal.

We also would call for research exploring the affective value of some of the other latent factors Jahoda mentioned – such as time structure, social contact, and activity – using real-time assessments [9]. Studies comparing employed and unemployed individuals support the role of these factors in explaining differences in psychological well-being, some of which even suggest the dominance of these factors relative to financial ones [78]. However, ESM-type studies examining these factors are lacking. For instance, studies could try to capture factors like cognitive busyness and the degree to which one’s workday is structured on a recurring (e.g., daily or within-day) basis to examine which of these various factors may yield positive affect. Again, to the degree that there is a tendency to under-appreciate the potential emotional value of these everyday latent factors, people may hold more negative views of their jobs than the actual experience of work warrants. Also, thought-sampling studies in which employees actually report about what they are thinking during the work day could complement real-time affect reports to provide a fuller picture of the impact of work context and specific activities (or lack thereof) on employee subjective experience.

Additional moderator results that warrant discussion are those concerning the nature of affect. First, affect intensity did not impact the pattern of results. Thus, while negative affect is somewhat more common in work than non-work, it is not especially more intense in the workplace. To the degree that statements about contexts being more or less “emotional” connote the intensity of emotions, this finding has theoretical significance for the emotionality of work. Specifically, this null finding seems to refute any characterization of modern workplaces as bureaucratic “iron cages” devoid of emotion [79] but also runs counter to the depiction of work as especially emotional. Rather, the intensity of emotions in the workplace and while working appears on par with emotional intensity in other contexts and activities.

In contrast to these null effects, the analyses yielded several differences among specific types of affect. In terms of positive affect, “happiness” and “enjoyment” were higher when people were not engaging in, or located at, work. These represent hedonic states and would seem to capture what is usually meant by “feeling good.” In contrast, “alert”, “interested”, and “cheerful” were higher for work than non-work. These latter findings generally seem consistent with the proposition that work is psychologically beneficial in part because it facilitates (mental) activity/engagement [9, 11]. Perhaps with the exception of the cheerfulness finding, the results also provide indirect support for the importance of work in fostering a sense of competence and meaningfulness [12]. In general, then, work possibly fosters more eudaimonic well-being, while non-work fosters hedonic well-being. This conclusion partially may explain why, on average, individuals who work are happier than those who do not work, even after accounting for the financial benefits of work [80]. Work and non-work experiences both can foster positive affect, but they provide different forms of it – this idea is consistent with our null finding for positive affect, more generally.

In contrast to the mixed findings for different types of PA, average levels of various specific types of NA generally were higher at work. The consistency across these many different negative moods and emotions implies that various workplace stressors and events are operative. That any one type of event could generate these somewhat heterogeneous forms of NA seems unlikely. Also, worth noting, though, is that these effect sizes - like those for generalized NA - generally were rather modest in magnitude.

In addition to these moderator results, a few other results from this study bear mention. One unexpected finding was that neither job complexity nor educational attainment impacted the effects. We certainly acknowledge that these results are tentative given methodological considerations. In particular, a limited number of studies provided data for us to calculate these two variables, thereby limiting the statistical power to detect a moderator effect. Moreover, in most cases, the samples were heterogenous on these factors, leading us to calculate average values for the sample. These caveats notwithstanding, the results do suggest that affective experiences may not vary as dramatically as other aspects of well-being between work and non-work domains. This may be due to affect largely being a result of discrete events rather than stable job characteristics [81].

Other empirical support would seem to support this notion. For example, in a large-scale meta-analysis of job features, the relationship between Hackman and Oldham’s job characteristics and anxiety (an affective state) were non-significant and/or close to zero in all cases with the exception of that involving feedback from the job [82]. Moreover, although some individual studies do report significant correlations, as others have noted [83], some of the most well-known studies linking job characteristics to affective states [42] have relied on self-reports of both constructs – thereby potentially yielding misleading results.

Rather than deriving from characteristics of the job, affect at work largely seems to result from particular events. Consistent with a main tenet of Affective Events Theory [81], studies link specific events to affective reactions, and especially interpersonal events (e.g., interactions with one’s manager, coworkers, clients; [84]). Thus, insofar as the nature and/or quality of social relations and interactions do not vary much as a function job complexity, affect may not vary greatly either. Certainly, though, we acknowledge that this explanation is tentative and requires empirical investigation.

With regard to demographic characteristics, we explored whether age, gender, and marital status functioned as moderators. We did find that older samples reported higher relative PA at work, a result that seems to coincide with at least some other data showing that job satisfaction increases with age [85]. But, with the exception of age, none of the factors altered the main effects. These results should be interpreted with caution since variance in the analyses was somewhat restricted by the use of study-level estimates for heterogeneous samples. Still, the pattern of weak and non-significant findings is suggestive that demographic factors generally are not strong determinants of differences in work versus non-work affect.

Finally, we also examined several methodological factors. Here, too, the results were mostly null, with only measurement schedule producing a significant effect. Specifically, for PA (but not NA), relative work affect was lower when non-work measurements were taken both before and after work compared to when non-work affect was assessed only after work. In contrast with previous studies on the diurnal rhythms of a working day [86], this result tentatively suggests that PA before work is greater than after work. While we only can conjecture about this finding, one possibility is that PA was measured later in the evening in studies where PA was measured only after work. Because PA may drop dramatically later in the evening [41], those estimates may be somewhat lower than if PA was measured right after work - perhaps explaining these findings.

### Study limitations

While we have noted some limitations of this research in the discussion above, additional factors necessitate mention. First, we generally were not able to assess what people were actually *doing* either at work or away from work. Whether people are having lunch with colleagues versus interacting with angry customers almost certainly will tend to produce different affective reactions. Similarly, when away from work, engaging in a leisure activity with friends will tend to produce quite different emotional reactions than does engaging in housework [16, 25]. Arguably, several of the variables we explored may have captured some of the goings-on generally taking place in the two environments (e.g., job complexity, whether people were married/cohabitating or living alone). However, these indirect attempts aside, the data in our sample largely precluded examination of the actual behaviors in which people were engaged in the work and non-work environments. We also were unable to explore specific locations or interaction partners because studies rarely reported that data, especially not within the work domain. Because of the potential insights that could be offered by such additional information, we encourage researchers to collect and report more detailed information about affective experiences, and particularly those occurring in different work contexts (e.g., meetings, email, teamwork, decision-making).

Also, some of the analyses were not based on terribly large sample sizes (or numbers of studies). Of note, we contacted over 20 additional “app” developers or companies whose app collected mood data, requesting them to share data for the project. For understandable legal reasons, however, they were not able to furnish the requested data. As described in the Method section, we also contacted hundreds of researchers, requesting data that we could have included in the analyses. Unfortunately, only about 10% of those researchers did provide such data. At the same time, we certainly wish to acknowledge and thank those who did so. As technologies continue emerging and greater quantities of data are collected, we are confident that larger data sets to further test ideas like the current ones will be available in the near future.

## Conclusion

The current study sheds light on individuals’ emotional experiences of work as compared to non-work. The results suggest that mean-level positive and negative affect at work do not differ dramatically from the levels we experience outside of our jobs. We hope the current study serves to stimulate future efforts to tease out some of the ideas proposed here as well as other ideas that may help elucidate these meta-analytic findings.

## Supporting information

S1 Appendix References for the meta-analysis database. (DOCX)

S1 Table Details on all primary studies included in the meta-analysis. (XLSX)
