# Supplementary material for: Just how miserable is work? A meta-analysis comparing work and non-work affect
Source: PLoS One. 2019 Mar 5;14(3):e0212594. doi: 10.1371/journal.pone.0212594 (PMC6400410; doi:10.1371/journal.pone.0212594)
Supplement: S1 Appendix — (DOCX) [file pone.0212594.s001.docx]

**S1 Appendix.** **References for the Meta-Analysis Database**

**Reference Section**

1. Auerbach, MA. The dynamic cycle of external, task interruptions: An ESM study of multiple role management. Doctoral dissertation, State University of New York at Albany. 2002. Available from ProQuest Dissertations and Theses database. (UMI No. 3053595).
2. Ayuso-Mateos, JL, Miret, M, Caballero, FF, Olaya, B, Haro, JM, Kowal, P, Chatterji, S. Multi-country evaluation of affective experience: Validation of an abbreviated version of the day reconstruction method in seven countries. PLoS ONE. 2013; 8(4), e61534. <http://doi.org/10.1371/journal.pone.0061534>
3. Barge-Schaapveld, DQCM, Nicolson, NA, Berkhof, J, deVries, MW. Quality of life in depression: Daily life determinants and variability. Psychiatry Research. 1999; 88(3), 173–189. <http://doi.org/10.1016/S0165-1781(99)00081-5>
4. Bernstein, JH. Waiting for the weekend: The role of autonomy, relatedness, competence and work experiences in patterns of weekly well-being*.* Doctoral dissertation, University of Rochester. 2004. Available from ProQuest Dissertations and Theses database. (UMI No. 3142281).
5. Brinton, JE. Strain-based work-home conflict: Examining the relative contribution of exhaustion and negative affect in the association between work demands and home behaviors. Masters Thesis, Wake Forest University. 2014. Available from ProQuest Dissertations and Theses database. (UMI No. 1561744).
6. Bryson, A, MacKerron, G. Are you happy while you work? The Economic Journal*.* 2017; 127(599), 106-125.
7. Bureau of Labor Statistics. American Time Use Survey 2010, 2012, and 2013 Multi-Year Well-Being Module Microdata Files [Data File].  2014. Available from: <http://www.bls.gov/tus/wbdatafiles_1013.htm>
8. Cao H. Work-family interface and outcomes: Testing the matching-domain hypothesis in Chinese sample. Doctoral dissertation, The Chinese University of Hong Kong. 2011. Available from ProQuest Dissertations and Theses database. (UMI No. 3531163).
9. Collip, D. Healthy control subjects from study of psychotic disorders. 2016; Unpublished raw data.
10. Collip, D, Nicolson, NA, Lardinois, M, Lataster, T, van Os, J, Myin-Germeys, I. Daily cortisol, stress reactivity and psychotic experiences in individuals at above average genetic risk for psychosis. *Psychological Medicine*. 2011; 41(11), 2305–2315. <http://doi.org/10.1017/S0033291711000602>
11. Damaske, S, Smyth, JM, Zawadzki, MJ. Has work replaced home as a haven? Re-examining Arlie Hochschild’s time bind proposition with objective stress data. Social Science & Medicine. 2014; 115, 130–138. <http://doi.org/10.1016/j.socscimed.2014.04.047>
12. Dudenhöffer, S, Dormann, C. Customer-related social stressors and service providers’ affective reactions. Journal of Organizational Behavior, 2013; 34(4), 520–539. <http://doi.org/10.1002/job.1826>
13. Eatough, EM, Meier, LL, Igic, I, Elfering, A, Spector, PE, Semmer, NK. You want me to do what? Two daily diary studies of illegitimate tasks and employee well-being. Journal of Organizational Behavior. 2015; 37(1), 108–127. <http://doi.org/10.1002/job.2032>
14. Fave, A. D., Massimini, F. Parenthood and the quality of experience in daily life: A longitudinal study. Social Indicators Research. 2004; 67(1/2), 75–106. <http://doi.org/10.1023/B:SOCI.0000007335.26602.59>
15. Ferguson, SG. Study of smokers. 2016. Unpublished raw data.
16. Fifield, J, Mcquillan, J, Armeli, S, Tennen, H, Reisine, S, Affleck, G. Chronic strain, daily work stress and pain among workers with rheumatoid arthritis: Does job stress make a bad day worse? Work & Stress. 2004; 18(4), 275–291. <http://doi.org/10.1080/02678370412331324996>
17. Houtveen, JH, Van Doornen, LJP. Negative affect and 24-hour ambulatory physiological recordings as predictors of spontaneous improvement of medically unexplained symptoms. Scandinavian Journal of Psychology. 2008; 49(6), 591–601. <http://doi.org/10.1111/j.1467-9450.2008.00684.x>
18. Ilies, R, Schwind, KM, Wagner, DT, Johnson, MD, DeRue, DS, Ilgen, DR. When can employees have a family life? The effects of daily workload and affect on work-family conflict and social behaviors at home. Journal of Applied Psychology. 2007; 92(5), 1368–1379. <http://doi.org/10.1037/0021-9010.92.5.1368>
19. Jackowska, M, Dockray, S, Endrighi, R, Hendrickx, H, Steptoe, A. Sleep problems and heart rate variability over the working day. Journal of Sleep Research. 2012; 21(4), 434–440. <http://doi.org/10.1111/j.1365-2869.2012.00996.x>
20. Jacobs, N, Myin-Germeys, I, Derom, C, Delespaul, P, van Os, J, Nicolson, NA. A momentary assessment study of the relationship between affective and adrenocortical stress responses in daily life. Biological Psychology. 2007; 74(1), 60–66. <http://doi.org/10.1016/j.biopsycho.2006.07.002>
21. Judge, TA, Ilies, R. Affect and job satisfaction: A study of their relationship at work and at home. Journal of Applied Psychology. 2004; 89*,* 661–673*.* <http://doi.org/10.1037/0021-9010.89.4.661>
22. Juster, RP, Moskowitz, DS, Lavoie, J, D’Antono, B. Sex-specific interaction effects of age, occupational status, and workplace stress on psychiatric symptoms and allostatic load among healthy Montreal workers. Stress. 2013; 16(6), 616–629. <http://doi.org/10.3109/10253890.2013.835395>
23. Kushlev, K, Dunn, EW, Ashton-James, CE. Does affluence impoverish the experience of parenting? Journal of Experimental Social Psychology. 2012; 48(6), 1381–1384. <http://doi.org/10.1016/j.jesp.2012.06.001>
24. Larson, R, Verma, S, Dworkin, J. Men’s work and family lives in India: The daily organization of time and emotion. Journal of Family Psychology. 2001; 15(2), 206–224. <http://doi.org/10.1037//0893-3200.15.2.206>
25. Lundberg, U, Dohns, IE, Melin, B, Sandsjö, L, Palmerud, G, Kadefors, R, Ekström, M, Parr, D. (1999). Psychophysiological stress responses, muscle tension, and neck and shoulder pain among supermarket cashiers. Journal of Occupational Health Psychology. 1999; 4(3), 245 -255. <http://doi.org/10.1037/1076-8998.4.3.245>
26. Meier, LL, Cho, E, Dumani, S. The effect of positive work reflection during leisure time on affective well-being: Results from three diary studies. Journal of Organizational Behavior. 2016; 37(2), 255–278. <http://doi.org/10.1002/job.2039>
27. Mellor-Marsá, B, Miret, M, Abad, FJ, Chatterji, S, Olaya, B, Tobiasz-Adamczyk, B, Caballero, FF. Measurement invariance of the day reconstruction method: Results from the COURAGE in Europe project. Journal of Happiness Studies. 2015; 17(5), 1769-1787. <http://doi.org/10.1007/s10902-015-9669-x>
28. Myin-Germeys, I, van Os, J, Schwartz, JE, Stone, AA, Delespaul, PA. Emotional reactivity to daily life stress in psychosis. Archives of General Psychiatry. 2001; 58(12), 1137–1144. <http://doi.org/10.1001/archpsyc.58.12.1137>
29. Myrtek, M., Fichtler, A., Strittmatter, M., Brügner, G. (1999). Stress and strain of blue and white collar workers during work and leisure time: Results of psychophysiological and behavioral monitoring. *Applied Ergonomics*, *30*(4), 341–351. <http://doi.org/10.1016/S0003-6870(98)00031-3>
30. Nagy, T, Salavecz, G, Simor, P, Purebl, G, Bódizs, R, Dockray, S, Steptoe, A. Frequent nightmares are associated with blunted cortisol awakening response in women. Physiology & Behavior. 2015; 147, 233–237. <http://doi.org/10.1016/j.physbeh.2015.05.001>
31. Oishi, S, Kurtz, JL, Miao, FF, Park, J, Whitchurch, E. (2011). The role of familiarity in daily well-being: Developmental and cultural variation. Developmental Psychology. 2011; 47(6), 1750–1756. <http://doi.org/10.1037/a0025305>
32. Peeters, F, Nicholson, NA, Berkhof, J. Cortisol responses to daily events in major depressive disorder. Psychosomatic Medicine. 2003; 65(5), 836–841. <http://doi.org/10.1097/01.PSY.0000088594.17747.2E>
33. Plemmons, SA. Recovery experiences: The importance of activities with regulatory rest. Doctoral dissertation, Purdue University. 2012. Available from ProQuest Dissertations and Theses database. (UMI No. 3544344).
34. Randall, AK, Schoebi, D. Lean on me: Susceptibility to partner affect attenuates psychological distress over a 12-month period. Emotion. 2015; 15(2), 201–210. <http://doi.org/10.1037/emo0000043>
35. Riediger, M, Schmiedek, F, Wagner, GG, Lindenberger, U. Seeking pleasure and seeking pain: Differences in prohedonic and contra-hedonic motivation from adolescence to old age. Psychological Science, 2009; 20(12), 1529–1535. <http://doi.org/10.1111/j.1467-9280.2009.02473.x>
36. Riediger, M, Wrzus, C, Wagner, GG. Happiness is pleasant, or is it? Implicit representations of affect valence are associated with contrahedonic motivation and mixed affect in daily life. Emotion. 2014; 14(5), 950–961. <http://doi.org/10.1037/a0037711>
37. Rodríguez-Sánchez, AM, Schaufeli, W, Salanova, M, Cifre, E, Sonnenschein, M. Enjoyment and absorption: An electronic diary study on daily flow patterns. Work & Stress. 2011; 25(1), 75–92. <http://doi.org/10.1080/02678373.2011.565619>
38. Schwerdtfeger, A, Konermann, L, Schönhofen, K. Self-efficacy as a health-protective resource in teachers? A biopsychological approach. Health Psychology. 2008; 27(3), 358–368. <http://doi.org/10.1037/0278-6133.27.3.358>
39. Smyth, JM, Zawadzki, MJ, Santuzzi, AM, Filipkowski, KB. Examining the effects of perceived social support on momentary mood and symptom reports in asthma and arthritis patients. Psychology & Health. 2014; 29(7), 813–831.
40. Snir, R, Zohar, D. Workaholism as discretionary time investment at work: An experience-sampling study. Applied Psychology. 2008; 57(1), 109–127. <http://doi.org/10.1111/j.1464-0597.2006.00270.x>
41. Song, Z, Foo, MD, & Uy, MA. Mood spillover and crossover among dual-earner couples: A cell phone event sampling study. Journal of Applied Psychology. 2008; 93(2), 443–452. <http://doi.org/10.1037/0021-9010.93.2.443>
42. Sonnentag, S, Binnewies, C. Daily affect spillover from work to home: Detachment from work and sleep as moderators. Journal of Vocational Behavior. 2013. 83(2), 198–208. <http://doi.org/10.1016/j.jvb.2013.03.008>
43. Stone, AA, Schwartz, JE, Schwarz, N, Schkade, D, Krueger, A, Kahneman, D. A population approach to the study of emotion: Diurnal rhythms of a working day examined with the day reconstruction method. Emotion. 2006; 6(1), 139–149. <http://doi.org/10.1037/1528-3542.6.1.139>
44. Thewissen, V, Bentall, RP, Lecomte, T, van Os, J, Myin-Germeys, I. Fluctuations in self-esteem and paranoia in the context of daily life. Journal of Abnormal Psychology. 2008; 117(1), 143–153. <http://doi.org/10.1037/0021-843X.117.1.143>
45. Van der Steen, J. T. Healthy control subjects from study of psychotic episodes. 2016. Unpublished raw data.
46. van Eck, M, Nicolson, NA, Berkhof, J. Effects of stressful daily events on mood states: Relationship to global perceived stress. Journal of Personality and Social Psychology*.* 1998. 75(6), 1572–1585. <http://doi.org/10.1037/0022-3514.75.6.1572>
47. van Hooff, MLM, Geurts, SAE. Need satisfaction during free evening hours: Examining its role in daily recovery. Stress and Health. 2014; 30(3), 198–208. <http://doi.org/10.1002/smi.2595>
48. van Hooff, MLM, Geurts, SAE. Need satisfaction and employees’ recovery state at work: A daily diary study. Journal of Occupational Health Psychology. 2015; *20*(3), 377–387. <http://doi.org/10.1037/a0038761>
49. van Hooff, MLM. The daily commute from work to home: Examining employees’ experiences in relation to their recovery status. Stress and Health. 2015; 31(2), 124–137. <http://doi.org/10.1002/smi.2534>
50. van Hooff, MLM, Geurts, SAE, Beckers, DGJ, Kompier, MAJ. Daily recovery from work: The role of activities, effort and pleasure. Work & Stress. 2011; *25*(1), 55–74. <http://doi.org/10.1080/02678373.2011.570941>
51. Verkuil, B, Brosschot, JF, Marques, AH, Kampschroer, K, Sternberg, EM, Thayer, JF. Gender differences in the impact of daily sadness on 24-h heart rate variability: Gender, sadness, and HRV. Psychophysiology. 2015; 52(12), 1682–1688. <http://doi.org/10.1111/psyp.12541>
52. Vincent, PC. The effects of daily work stress on employed mothers’ mood states. Doctoral dissertation, University of California, Los Angeles. 1999. Available from ProQuest Dissertations and Theses database. (UMI No. 9939070).
53. Waite, LJ, Schneider, B. (1997). *Contemporary families and experiences of work: A proposal for a New Alfred P. Sloan Center of Working Families.* Proposal submitted to the Alfred P. Sloan Center.
54. White, MP, Dolan, P. Accounting for the richness of daily activities. Psychological Science. 2009; 20(8), 1000–1008. <http://doi.org/10.1111/j.1467-9280.2009.02392.x>
55. Williams, KJ, & Alliger, GM. Role stressors, mood spillover, and perceptions of work-family conflict in employed parents. Academy of Management Journal. 1994; 37(4), 837–868. <http://doi.org/10.2307/256602>
56. Wrzus, C, Müller, V, Wagner, GG, Lindenberger, U, Riediger, M. Affective and cardiovascular responding to unpleasant events from adolescence to old age: Complexity of events matters. Developmental Psychology. 2013; 49(2), 384–397. <http://doi.org/10.1037/a0028325>
57. Wrzus, C, Wagner, GG, Riediger, M. Feeling good when sleeping in? Day-to-day associations between sleep duration and affective well-being differ from youth to old age. Emotion. 2014; 14(3), 624–628. <http://doi.org/10.1037/a0035349>
58. Zawadzki, MJ, Smyth, JM, Costigan, HJ. Real-time associations between engaging in leisure and daily health and well-being. Annals of Behavioral Medicine. 2015; 49(4), 605–615. <http://doi.org/10.1007/s12160-015-9694-3>
59. Zhou, ZE, Yan, Y, Che, XX, Meier, LL. Effect of workplace incivility on end-of-work negative affect: Examining individual and organizational moderators in a daily diary study. Journal of Occupational Health Psychology. 2015; 20(1), 117–130. <http://doi.org/10.1037/a0038167>
